# Polypharmacy Thresholds that Best Predict Emergency Room Visits and Mortality in Older Adults: A Population-Based Study in Québec, Canada

**DOI:** 10.1177/07334648251391524

**Published:** 2025-10-28

**Authors:** Alexandre Campeau Calfat, Justin P. Turner, Marc Simard, Marjolaine Dubé, Caroline Sirois

**Affiliations:** 1 Faculty of Pharmacy, 70346 Université Laval, Québec City, QC, Canada; 2 54470 Institut national de santé publique du Québec, QC, Canada; 3 607356 VITAM – Centre de Recherche en Santé Durable, Québec City, QC, Canada; 4 Centre for Medicine Use and Safety, Faculty of Pharmacy and Pharmaceutical Science, Monash University, Melbourne, VIC, Australia

**Keywords:** polypharmacy, health outcomes, pharmacoepidemiology, medication

## Abstract

**Background:**

Polypharmacy is prevalent among older adults. Identifying informative medication thresholds could enhance clinical decision-making and healthcare planning.

**Objectives:**

To measure the predictive capacity of medication count and polypharmacy thresholds on (1) frequent emergency visits and (2) mortality, in community-dwelling older adults in Quebec, Canada; To measure the predictive capacity of inappropriateness criteria (potentially inappropriate medications, anticholinergic burden level, and drug–drug interactions) on these same outcomes.

**Methods:**

We conducted a population-based study using the Quebec Integrated Chronic Disease Surveillance System. Using multivariable logistic regression models, we assessed the predictive capacity [area under the curve (AUC)] of medication use (medication count, polypharmacy thresholds, number of inappropriateness criteria) on frequent emergency visits (≥3) and mortality from April 2, 2022, to March 31, 2023. Findings were interpreted from two perspectives: clinical (with models including individuals’ clinical characteristics, such as chronic diseases) and public health (with models excluding clinical characteristics to mimic polypharmacy measures usually used in surveillance).

**Results:**

Medication count remained the most informative predictor in the clinical perspective analyses, with AUC = 0.739 for frequent emergency visits and AUC = 0.804 for one-year mortality, while medication thresholds lacked discrimination. Public health perspective analyses resulted in a bell-shaped predictive pattern peaking at eight medications for both frequent emergency visits (AUC = 0.690) and one-year mortality (AUC = 0.764). Inappropriateness criteria did not outperform medication count or polypharmacy thresholds.

**Conclusion:**

From a clinical perspective, medication count is more informative than polypharmacy thresholds. From a public health perspective, a threshold of eight medications may serve as a useful polypharmacy measure.

**What this paper adds**

- Medication count alone can outperform complex inappropriateness criteria in predicting key health outcomes.
- Eight medications seems to be an optimal threshold for identifying higher-risk older adults.

**Applications of study findings**

- The eight-medication threshold offers a practical research definition of potentially inappropriate polypharmacy.
- This threshold provides a public health metric to better target interventions.
- Clinicians should reassess medication needs as older patients experience increased medication use.

## Introduction

The use of multiple medications, commonly referred to as polypharmacy, is increasingly prevalent, particularly among older adults (Gosselin et al., 2020). In 2022, 47% of community-dwelling older adults in Quebec, Canada, used at least 5 medications (Campeau Calfat et al., 2024). As the number of medications increases, so does the risk of medication-related problems, including adverse drug events, and increased healthcare utilization (Chae et al., 2024; Fried et al., 2014). Polypharmacy has been strongly associated with the use of inappropriateness criteria such as potentially inappropriate medications (PIMs), anticholinergic burden, and drug–drug interactions, all of which can contribute to poor health outcomes (Campeau Calfat et al., 2024). Although the most common definition of polypharmacy is the use of five or more medications, there remains uncertainty regarding the optimal cutoff to define polypharmacy (Sirois et al., 2019).

Several studies have sought to determine medication thresholds that best predict health risks. For instance, predictive models have identified threshold of 3.5, 4.5, 5.5, and 6.5 medications for identifying cognitive impairment, mortality, disability, and frailty, respectively, in a cohort of community-dwelling older males (Gnjidic et al., 2012). Other studies have consistently shown that as medication use increases, so does the risk of adverse outcomes, such as mortality (Boateng et al., 2025; Chae et al., 2024; Davies et al., 2022; Leelakanok et al., 2017) and emergency department visits (Chae et al., 2024; Fried et al., 2014; Park et al., 2023). This suggests that the relationship between polypharmacy and health risks may not be defined by a single universal threshold but rather by a spectrum of risk that varies across populations and specific health outcomes.

Despite these findings, medication use is often treated as a dichotomous variable (Davies et al., 2022; Fried et al., 2014; Leelakanok et al., 2017; Turner et al., 2016) or categorized into broad subgroups (e.g., 1–4, 5–9, and ≥10 medications) (Chae et al., 2024; Fried et al., 2014; Leelakanok et al., 2017; Park et al., 2022). These approaches may oversimplify the association between medication burden and outcomes, potentially missing important nuances. For instance, thresholds that accurately predict one outcome, such as mortality, may differ from those relevant to predicting healthcare utilization. However, few studies have systematically examined the predictive capacity of medication count across all possible thresholds, particularly in a population-based setting. Similarly, few studies have been interested in the number of inappropriateness criteria such as PIMs, anticholinergic burden, and drug–drug interactions (Campeau Calfat et al., 2024).

Thus, we aimed to identify polypharmacy and inappropriateness criteria thresholds that most effectively predict health outcomes. Our approach integrated two complementary perspectives: clinical and public health. The clinical perspective, which incorporates individual-level clinical data such as the presence of chronic diseases, emphasizes personalized risk prediction to inform comprehensive care. In contrast, the public health perspective considers polypharmacy as a measure for population-level surveillance and, by design, only accounts for a few individual-level clinical data. By combining these perspectives, we strive to enhance the applicability and relevance of our findings across diverse healthcare contexts.

From both clinical and public health perspectives, our study aimed to assess the predictive capacity of medication count and polypharmacy thresholds on two key outcomes: (1) frequent emergency visits and (2) mortality, in community-dwelling older adults in Quebec, Canada. We also assessed the predictive capacity of the number of inappropriateness criteria met, specifically PIMs, anticholinergic burden level, and drug–drug interactions, on these same outcomes. Frequent emergency visits and mortality were selected because they reflect distinct but complementary challenges in older populations. Frequent emergency department users often present with complex health needs, including chronic diseases and social vulnerabilities. They are often frequent users of other healthcare services, making them key target for both clinical and public health interventions (LaCalle & Rabin, 2010; Slankamenac et al., 2019). Mortality, in turn, is a universal and easily measurable outcome that is relevant to both individual care and population health monitoring. Both outcomes are available in health administrative databases and provide meaningful tools to assess health risk and healthcare burden.

## Methods

### Data Source

We conducted a population-based study using the Quebec Integrated Chronic Disease Surveillance System (QICDSS), managed by the Québec National Institute of Public Health [Institut national de santé publique du Québec (INSPQ)] (Blais et al., 2014). The QICDSS, with data available since 1996, integrates information from five key databases under a unique anonymized identification number. These databases include the health insurance registry, which provides sociodemographic data; the MED-ÉCHO database, which contains hospitalization records; the vital statistics death database, which includes dates of death and associated causes; the physician claims database, which records all fee-for-service billings; and the pharmaceutical services database, which details prescription medication claims under the public drug plan. The QICDSS covers approximately 99% of the population in Quebec, and about 90% of individuals aged 65 and older are covered under the public drug plan (Blais et al., 2014). The reporting of this study conforms to the STROBE statement (von Elm et al., 2007).

### Study Population

We included all community-dwelling individuals aged over 65 who were enrolled in the public drug plan as of April 1, 2022. To be included, individuals needed an enrollment in the public drug plan from October 1, 2021, to April 1, 2022. A six-month lookback period was applied to account for medications dispensed less frequently, such as denosumab. Individuals living in long-term care facilities or who were hospitalized on April 1, 2022 were excluded, as their medication use in those settings is not recorded in the QICDSS.

For analyses assessing frequent emergency visits, participants were required to be alive and continuously covered by the public health plan through the year following the index date (April 1,2022) to ensure that individuals were community-dwelling throughout the period, and to minimize competing risks (e.g., death). However, for analyses of one-year mortality, this requirement was not applicable, as death inherently terminates public health coverage.

### Outcomes

(1) Frequent emergency visits were defined as the presence of 3 or more emergency visits between April 2, 2022, and March 31, 2023, as recorded in the health insurance registry. This corresponds to the 95th percentile of the number of visits per year in Québec (Gaulin et al., 2019). Commonly used to define frequent emergency visits (LaCalle & Rabin, 2010), this threshold has been previously validated in the QICDSS (Gaulin et al., 2019; Simard et al., 2024). A single visit to the emergency department was defined as one or more emergency department related claims on up to two consecutive days (Gaulin et al., 2019).
(2) One-year mortality was ascertained using the vital statistics death database. Every death that occurred between April 2, 2022, and March 31, 2023, was identified in the database.

### Medication Use at Index Date (April 1, 2022)

#### Medication Count and Polypharmacy Thresholds

We calculated the total number of current medications on April 1, 2022. A medication was classified as current if its treatment duration, as recorded by the pharmacist at dispensing, included April 1, 2022. The common denomination code, equivalent to the 5th level of the Anatomical Therapeutic Chemical (ATC) classification, was used to identify current medications in the QICDSS pharmaceutical services database. Medications with multiple active ingredients were assigned a single code (e.g., a tablet containing two active ingredients was counted as one medication). All medication forms, including oral, topical, and inhaled, were considered. Codes corresponding to devices, equipment, and supplies, as well as antiseptic, solvents, dressings, reagents, and any other codes not referring to active pharmaceutical ingredients were systematically excluded from the medication counts. Individuals were categorized based on the number of current medications they had on April 1, 2022, with specific categories for each number (1, 2, …, ≥20). For analysis purposes, those using ≥20 medications were grouped together, as they accounted for less than 0.5% of the population. Polypharmacy thresholds were calculated by aggregating all users of the medication who met or exceeded the defined threshold.

#### Potentially Inappropriate Medications, Anticholinergic Burden, and Drug–Drug Interactions

PIMs were identified using the 2019 update of the American Geriatrics Society Beers Criteria (American Geriatrics Society, 2019), which are commonly applied in studies using administrative data due to their minimal clinical data requirements (Gosselin et al., 2023; Mekonnen et al., 2021; Mohamed et al., 2020). The Beers Criteria were adapted to reflect medications available in Canada and the data in the QICDSS (Gosselin et al., 2023). Any medication listed in Table 2 of the Beers Criteria was considered a PIM, except for doxepin and digoxin, which were excluded from our study because we did not consider dosage information. Medications subject to conditional classification, including proton pump inhibitors, insulin, and antipsychotics, were considered PIMs only when the conditions specified in the Beers criteria were met. As per previous analysis of Québec data (Campeau Calfat et al., 2024), aspirin was classified as a PIM when used for primary prevention, according to the 2023 update of the Beers Criteria (American Geriatrics Society, 2023). To determine whether aspirin was used for secondary prevention, we relied on diagnostic codes related to coronary events, heart failure, and cerebrovascular or vascular diseases. This approach was specific to aspirin, as significant changes in recommendations between the 2019 and 2023 Beers Criteria (American Geriatrics Society, 2019, 2023) were made based on new research and updated guidelines regarding its use in primary prevention. The total number of PIMs per individual was calculated, counting each medication once, even if listed multiple times in the Beers Criteria.

The anticholinergic burden was measured using the Anticholinergic Cognitive Burden (ACB) scale, a validated tool for assessing the risk of cognitive decline in older adults (Srikartika et al., 2025). Each medication was assigned an ACB score ranging from 0 to 3 (Boustani et al., 2008), and the scores were summed for each individual based on their claimed medications. Higher ACB levels indicate a greater anticholinergic burden, associated with an increased risk of cognitive impairment and other adverse effects (Boustani et al., 2008).

Clinically significant drug–drug interactions were identified using the medication combinations listed in Table 5 of the 2019 Beers Criteria (American Geriatrics Society, 2019). The total number of drug–drug interactions for each individual was then calculated.

#### Covariates

Age, sex, social and material deprivation index, area of residence (urban vs rural), and disease count (for the clinical perspective) were used as covariates in the predictive models. Age was categorized as follows: 66–70 years, 71–75 years, 76–80 years, 81–85 years, and 86 years and older. Deprivation indexes are validated proxies of socio-economic status based on postal code (Pampalon et al., 2004). The quality of social networks can be expressed by the social deprivation index, whereas the quality of material goods owned by the population studied can be gauged by the material deprivation index. The first quintile of both indexes includes the least deprived individuals while the fifth quintile contains the most deprived (Pampalon et al., 2004). Disease count was assessed using a five-year lookback period and categorized as follows: 0, 1, 2, 3, and ≥4 diseases. We considered the 31 diseases set in the combined comorbidity score, which has been validated for the Québec population (Simard et al., 2018).

### Statistical Analyses

Multivariable predictive logistic models were performed to predict both frequent emergency visits and one-year mortality outcomes. As opposed to explanatory models, predictive models do not aim to identify causal mechanisms (Sainani, 2014). They rather seek to predict an outcome based on a set of variables, and therefore classify at risk individuals (Sainani, 2014). Performance of each model was assessed using three measures (Steyerberg et al., 2010): (1) the discrimination capacity of each model, that is the ability to correctly identify patients having the outcome, was measured with the area under the receiver operating characteristic curve (AUC); (2) the overall performance of the model calculated with the scaled Brier score, which values range from 0 to 1 (higher value indicates better performance); and (3) the net reclassification improvement (NRI) that assesses the improvement from baseline in reclassification of individuals into different risk categories. All applicable hypotheses relevant to logistic regression were verified: (1) absence of multicollinearity among variables and (2) linearity of the logit for models including continuous variables. All 99% confidence intervals were obtained using a bootstrap resampling method.

#### Clinical Perspective

From a clinical perspective, the baseline model included all covariates, including disease count, as predictors. We included disease count since such information is often available in clinical practice. Medication count (1, 2, …, ≥20) and polypharmacy thresholds (≥2, …, ≥20) were independently added to this baseline model. Three other separate models were constructed, each incorporating the covariates from the baseline model along with one of the following continuous variables: number of PIMs, anticholinergic burden level, and number of drug–drug interactions. Odds ratios were calculated using 99% confidence interval (CI).

#### Public Health Perspective

From a public health perspective, the same models were constructed, excluding disease count, as it is not typically used as a stratification factor in public health measures of polypharmacy. The improvement in classification provided by adding a variable to the baseline models is particularly relevant from a public health perspective. It can help better identify people who are at a higher risk of outcomes and who could benefit from targeted health interventions. The percentage improvement for the outcome indicates how much better the model becomes at correctly identifying individuals who do have the outcome. The percentage improvement for those without the outcome shows how well the model avoids wrongly classifying people as at risk when they are not. Thus, this measure indicates whether the model improves at identifying the right individuals—both those who are at a risk and those who are not.

#### Ethics

The provincial Public Health Research Ethics Board and the Quebec Commission protecting access to information have approved the use of the QICDSS for surveillance purposes. No written informed consent was required.

## Results

**Figure 1.**
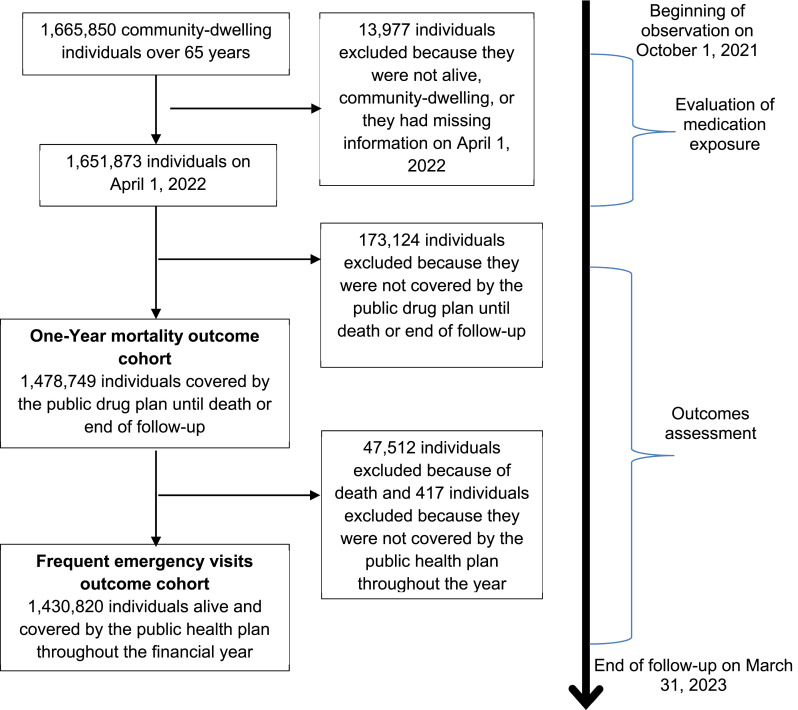
Flowchart of the Selection Process for Community-Dwelling Individuals Aged Over 65 years in Québec, Canada, on April 1, 2022

There were 1,665,850 community-dwelling individuals aged over 65 years on April 1, 2022, in Quebec. From those, 1,430,820 individuals were included in analysis used to predict frequent emergency visits and 1,478,749 were included in models used to predict one-year mortality (Figure 1). Included individuals had a mean number of 5 ± 4 medications (Table 1). Frequent emergency visits and mortality occurred in 4.3% and 3.2% of the population, respectively.

**Table 1.** Characteristics of community-dwelling adults aged over 65 years covered by the public drug plan in Quebec, Canada, on April 1, 2022

	Individuals included for each outcome			
	Frequent emergency visit		One-year mortality	
	*N* = 1,430,820		*N* = 1,478,749	
	*N*	%	*N*	%
Age, years [mean ± SD]	75.5 ± 7.0		75.7 ± 7.1	
Age, years				
66–70	459,639	32.1	465,444	31.5
71–75	391,386	27.4	398,782	27.0
76–80	278,639	19.5	287,330	19.4
81–85	165,711	11.6	174,327	11.8
≥86	135,445	9.5	152,866	10.3
Sex				
Female	783,808	54.8	807,988	54.6
Male	647,012	45.2	670,761	45.4
Social deprivation index				
1 (least deprived)	243,838	17.0	249,959	16.9
2	264,068	18.5	271,506	18.4
3	262,965	18.4	270,780	18.3
4	268,351	18.8	277,057	18.7
5 (most deprived)	258,117	18.0	267,275	18.1
Unknown	133,481	9.3	142,172	9.6
Material deprivation index				
1 (least deprived)	240,785	16.8	246,905	16.7
2	244,548	17.1	251,338	17.0
3	258,018	18.0	265,795	18.0
4	274,062	19.2	282,841	19.1
5 (most deprived)	279,926	19.6	289,698	19.6
Unknown	133,481	9.3	142,172	9.6
Area of residence				
Urban (10k inhabitants or more)	1,118,303	78.2	1,156,131	78.2
Rural (less than 10k inhabitants)	312,517	21.8	322,618	21.8
Disease count^a^, mean ± SD	1.7 ± 2.2		1.8 ± 2.4	
Number of chronic diseases				
0	525,018	36.7	531,093	35.9
1	381,709	26.7	388,459	26.3
2	192,622	13.5	197,962	13.4
3	106,931	7.5	111,429	7.5
≥4	224,540	15.7	249,806	16.9
Number of current medications, mean ± SD	4.9 ± 4.0		5.0 ± 4.1	
Number of current medications				
0	221,147	15.5	225,012	15.2
1–4	535,892	37.5	544,634	36.8
5–9	486,503	34.0	504,618	34.1
10–14	153,382	10.7	165,996	11.2
15–19	29,778	2.1	33,644	2.3
≥20	4,118	0.3	4,845	0.3
Number of potentially inappropriate medications, mean ± SD	0.6 ± 0.8		0.6 ± 0.8	
Number of potentially inappropriate medications				
0	803,136	56.1	821,190	55.5
1	475,063	33.2	495,098	33.5
2	122,386	8.6	130,137	8.8
≥3	30,235	2.1	32,324	2.2
Number of drug–drug interactions, mean ± SD	0.1 ± 0.3		0.1 ± 0.3	
Number of drug–drug interactions				
0	1,344,961	94.0	1,386,655	93.8
1	70,099	4.9	74,404	5.0
2	11,210	0.8	12,461	0.8
≥3	4,550	0.3	5,229	0.4
Level of anticholinergic burden, mean ± SD	0.6 ± 1.3		0.6 ± 1.3	
Level of anticholinergic burden				
0	1,008,974	70.5	1,029,941	69.6
1	217,498	15.2	229,351	15.5
2	61,336	4.3	66,864	4.5
≥3	143,012	10.0	152,593	10.3
Presence of outcome	62,081	4.3	47,512	3.2

SD: standard deviation.

^a^We considered the 31 disease set in the combined comorbidity score, i.e.: hypertension, chronic pulmonary disease, cardiac arrhythmias, diabetes (uncomplicated and complicated), anemia, any tumor without metastasis, hypothyroidism, renal disease, fluid and electrolyte disorders, peripheral vascular disorders, myocardial infarction, congestive heart failure, obesity, valvular disease, metastatic cancer, dementia, cerebrovascular disease, depression, neurological disorders, alcohol abuse, liver disease, psychoses, pulmonary circulation disorders, rheumatoid arthritis/collagen vascular disease, coagulopathy, weight loss, drug abuse, paralysis, ulcer disease, and AIDS/HIV.

### Clinical Perspective

Adding medication count to the baseline model resulted in improvement in predictive capacity for both outcomes. There was an increase in AUC of 0.01 and 0.004 from baseline for frequent emergency visits and one-year mortality, respectively (Table 2). For each added medication, the odds of frequent emergency visit increased by 8% (OR: 1.08; 99% CI 1.08–1.08) while the odds of one-year mortality increased by 6% (OR: 1.06; 99% CI 1.05-1.06) (Table S3).

**Table 2.** Performance measures of each model used to predict frequent emergency visits (≥3) and one-year mortality from a clinical perspective

				Reclassification improvement		
Outcomes	Model	Discrimination AUC (99% CI)	Overall performance Scaled Brier Score (99%CI)	NRI (99%CI)	Outcome percent improvement (%)	Non outcome percent improvement (%)
Frequent emergency visits (≥3)	Baseline	0.729 (0.726–0.731)	0.035 (0.033–0.036)	REF	REF	REF
	Threshold9+^a^	0.737 (0.733–0.739)	0.039 (0.037–0.040)	0.431 (0.420– 0.446)	42.1	79.5
	RxCount	0.739 (0.736–0.742)	0.042 (0.041–0.043)	0.272 (0.262–0.283)	52.9	60.7
	ACB	0.736 (0.733–0.738)	0.038 (0.037–0.039)	0.265 (0.256–0.274))	37.0	76.3
	PIMs	0.735 (0.732–0.737)	0.037 (0.035–0.038)	0.205 (0.196–0.214)	49.5	60.7
	Drug–drug interactions	0.734 (0.731–0.736)	0.038 (0.036–0.039)	0.123 (0.108–0.138)	20.1	86.0
One-year mortality	Baseline	0.800 (0.797–0.802)	0.052 (0.051–0.054)	REF	REF	REF
	Threshold9+^a^	0.804 (0.801–0.806)	0.054 (0.052–0.055)	0.455 (0.441–0.472)	44.3	78.5
	RxCount	0.804 (0.801–0.807)	0.055 (0.053–0.056)	0.249 (0.235–0.265)	52.3	60.2
	ACB	0.805 (0.803–0.808)	0.056 (0.054–0.057)	0.319 (0.308–0.331)	39.9	76.0
	PIMs	0.802 (0.799–0.805)	0.053 (0.051–0.054)	0.192 (0.174–0.222)	50.2	59.5
	Drug-drug interactions	0.804 (0.801–0.807)	0.054 (0.053–0.056)	0.191 (0.179–0.206)	20.3	89.3

CI: confidence interval; AUC: Area Under Curve; NRI: Net Reclassification Improvement; PIMs: potentially inappropriate medications; ACB: anticholinergic burden.

Predictors included in Baseline models: age, sex, social deprivation, material deprivation, zone, disease count.

Interpretation of discrimination: the ability to identify rightfully individuals who had the outcomes.

Interpretation of Scaled Brier Score: the overall performance of the models; higher value indicates better performance.

Interpretation of NRI: the improvement in classification provided by adding a variable to the baseline models; higher value indicates better reclassification. The outcome percent improvement indicates how much better the model is at correctly identifying at risk individuals. The nonoutcome percent improvement indicates the model’s ability to correctly identify individuals without the outcome, avoiding false positive.

^a^We presented the threshold of nine medications as it presents the best discrimination among all thresholds from a clinical perspective. Complete information on models using polypharmacy thresholds is presented in Tables S1 and S2.

**Figure 2.**
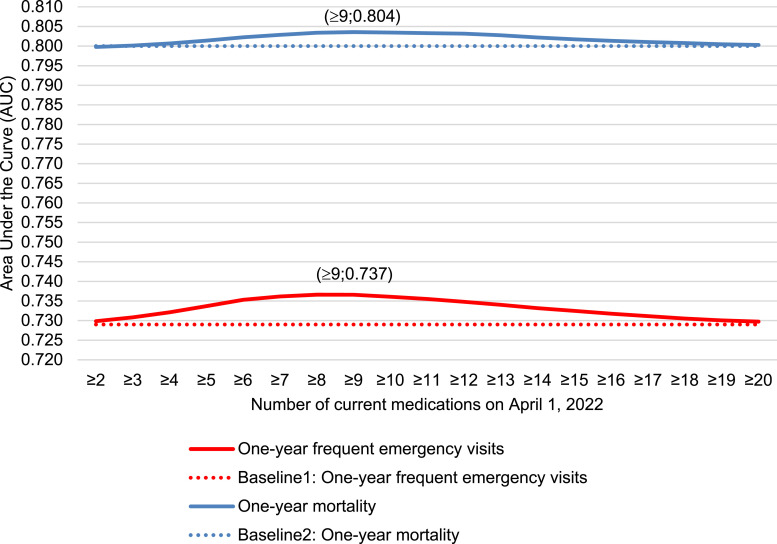
Area Under the Curve (AUC) of Predictive Models Using Polypharmacy Thresholds (≥2 to ≥20 Current Medications): A Clinical Perspective including Disease Count. Predictors included in Baseline1 and Baseline2 models: age, sex social deprivation, material deprivation, zone, disease count

Polypharmacy thresholds slightly improved the predictive capacity of the baseline model for both outcomes (Tables 2, S1, S2, and Figure 2). The maximum AUC change from baseline for frequent emergency visits and one-year mortality was 0.008 and 0.004, at a threshold of 9 medications (Table 2, S1, S2). Overall performances and NRI of each model followed a similar pattern, with higher thresholds of polypharmacy not yielding better performances than lower ones. An increase in odds ratios was observed with rising thresholds used to define polypharmacy (Table S3). At a threshold of nine medications, the odds ratios of frequent emergency visits and mortality were 1.75 (99% CI 1.71–1.80) and 1.51 (99% CI 1.47–1.56), respectively (Table S3).

Models that included inappropriateness criteria showed performance measures in a similar range to those observed with medication count. Anticholinergic burden offered the best discrimination capacity for both outcomes (AUC = 0.736 and 0.805 for frequent emergency visits and mortality, respectively). ACB levels were associated with a similar increase in odds for both outcomes, but with the lowest odds ratios compared to number of PIMs and drug–drug interactions (OR: 1.16, 99% CI: 1.15–1.16 for frequent emergency visits, and OR: 1.17, 99% CI: 1.16–1.18 for mortality). For PIMs, there were a 0.006 and 0.002 increase in AUC from baseline for both frequent emergency visits and mortality, respectively. The number of PIMs was associated with greater odds of frequent emergency visits (OR: 1.26, 99% CI: 1.25–1.28) than mortality (OR: 1.18, 99% CI: 1.16–1.20). For drug–drug interactions, discrimination capacity was improved by 0.005 and 0.004 from baseline for frequent emergency visits and mortality, respectively. Among all inappropriateness criteria, drug–drug interactions had the strongest association with both frequent emergency visits and mortality (OR 1.52; 99% CI 1.49–1.55 and 1.51; 99% CI 1.48–1.55, respectively).

### Public Health Perspective

Models using medication count resulted in improvement in the predictive capacity from baseline models for both outcomes. There was a 0.069 and 0.039 increase in AUC from baseline for frequent emergency visits and one-year mortality, respectively (Table 3). Classification of individuals with frequent and non-frequent emergency visits was improved by 57.2% and 66.7%, respectively, when medication count was added to the baseline model (NRI = 0.479; 99% CI 0.468–0.490). Similarly, the classification of individuals with mortality and non-mortality improved by 56.9% and 65.8%, respectively (Table 3).

**Table 3.** Performance measures of each model used to predict one-year frequent emergency visits (≥3) and one-year mortality from a public health perspective

				Reclassification improvement		
Outcomes	Model	Discrimination AUC (99% CI)	Overall performance Scaled Brier Score (99%CI)	NRI (99%CI)	Outcome percent improvement (%)	Non outcome percent improvement (%)
Frequent emergency visits (≥3)	Baseline	0.633 (0.630–0.636)	0.010 (0.010–0.011)	REF	REF	REF
	Threshold8+^a^	0.690 (0.687–0.693)	0.022 (0.021–0.023)	0.536 (0.526–0.547)	48.7	78.1
	RxCount	0.702 (0.700–0.706)	0.031 (0.029–0.032)	0.479 (0.468–0.490)	57.2	66.7
	ACB	0.672 (0.669–0.675)	0.017 (0.016–0.018)	0.364 (0.354–0.374)	43.0	75.2
	PIMs	0.666 (0.663–0.669)	0.015 (0.015–0.016)	0.375 (0.365–0.384)	61.6	57.1
	Drug-drug interactions	0.659 (0.656–0.662)	0.015 (0.015–0.016)	0.193 (0.186 – 0.200)	15.3	94.4
One-year mortality	Baseline	0.731 (0.728–0.735)	0.031 (0.030–0.032)	REF	REF	REF
	Threshold8+^a^	0.764 (0.761–0.767)	0.038 (0.037–0.039)	0.572 (0.560–0.585)	51.7	76.9
	RxCount	0.770 (0.768–0.774)	0.042 (0.041–0.044)	0.455 (0.441–0.471)	56.9	65.8
	ACB	0.757 (0.754–0.760)	0.037 (0.036–0.039)	0.417 (0.403–0.429)	45.1	75.8
	PIMs	0.746 (0.743–0.750)	0.033 (0.032–0.034)	0.368 (0.355–0.380)	62.0	56.4
	Drug–drug interactions	0.749 (0.746–0.752)	0.034 (0.033–0.035)	0.190 (0.181 – 0.198)	15.4	94.1

CI: confidence interval; AUC: Area Under Curve; NRI: Net Reclassification Improvement; PIM: potentially inappropriate medication; ACB: anticholinergic burden.

Predictors included in Baseline models: age, sex, social deprivation, material deprivation, zone.

Interpretation of discrimination: the ability to identify rightfully individuals who had the outcomes.

Interpretation of Scaled Brier Score: the overall performance of the models; higher value indicates better performance.

Interpretation of NRI: the improvement in classification provided by adding a variable to the baseline models; higher value indicates better reclassification. The outcome percent improvement indicates how much better the model is at correctly identifying at risk individuals. The nonoutcome percent improvement indicates the model’s ability to correctly identify individuals without the outcome, avoiding false positive.

^a^We presented the threshold of eight medications as it presents the best discrimination among all thresholds from a public health perspective. Complete information on models using polypharmacy thresholds is presented in Table 4 and Table S5.

**Figure 3.**
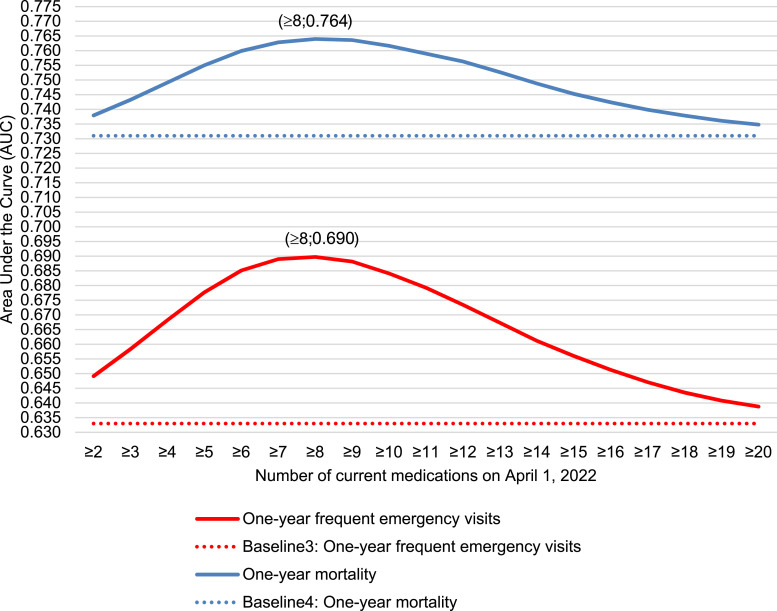
Area Under the Curve (AUC) of Predictive Models Using Polypharmacy Thresholds (≥2 to ≥20 Current Medications): A Public Health Perspective excluding Disease Count. Predictors included in Baseline3 and Baseline4 models: age, sex social deprivation, material deprivation, zone

The inclusion of polypharmacy thresholds in the baseline model resulted in a bell-shaped increase in AUC (Figure 3). The maximum change in AUC from baseline was 0.057 for frequent emergency visits and 0.033 for one-year mortality (Table 3 and Tables S4, S5). Adding the threshold of eight medications to the baseline model resulted in an NRI of 0.536 (99% CI 0.526–0.547), reflecting a 48.7% improvement in correctly reclassifying individuals who frequently visited the emergency, and a 78.1% improvement in correctly reclassifying individuals who did not (Table 3). This improvement was slightly lower than the one offered by using a threshold of seven medications (NRI = 0.557 99% CI 0.548–0.568) (Table S4). In regard to mortality, adding a threshold of eight medications to the baseline model resulted in an NRI of 0.572 (99% CI 0.560–0.585), reflecting a 51.7% improvement in correctly reclassifying individuals who died and a 76.9% improvement in correctly reclassifying individuals who survived (Table 3). A threshold of seven medications offered a better improvement (NRI = 0.596 99% CI 0.585–0.609) (Table S5).

Models that included inappropriateness criteria appeared to yield lower performance measures than those that incorporated medication count. Anticholinergic burden offered the best discrimination capacity for both outcomes when compared to the number of PIMs and drug–drug interactions. Percent improvement following the addition of inappropriateness criteria was similar for both outcomes (Table 3). Including either ACB level or drug–drug interactions in the baseline models substantially reduced the rate of false positives, compared to the improvement observed in correctly classifying individuals with the outcome. The inclusion of PIMs led to approximately a 60% improvement in correctly reclassifying individuals with and without the outcome (Table 3).

## Discussion

Including medication count improved the predictive performance of parsimonious models developed from both the clinical and public health perspectives for predicting frequent emergency department visits and mortality. When polypharmacy was modeled using thresholds, it also enhanced the performance of models developed from a public health perspective for both outcomes. From a clinical perspective, this improvement was modest, suggesting that strong predictors such as disease burden (Gaulin et al., 2019; Simard et al., 2024) may already capture much of the discrimination associated with medication use. From both perspectives, models using inappropriateness criteria did not outperform those based on medication count or polypharmacy thresholds. All medication-related measures improved the predictive performance of the models, underscoring the importance of considering these measures when using administrative databases to better understand predictors of both outcomes in older adults.

### Implication

From a clinical perspective, medication count could serve as a proxy for predicting individualized risk of frequent emergency visits and one-year mortality. When compared to polypharmacy thresholds, medication count offers the highest discrimination capacity. Thus, from a clinical perspective, each added medication contributes meaningfully to assessing an individual’s risk.

From a public health perspective, a bell-shaped predictive pattern emerged, with peak discrimination observed at a threshold of eight medications. This suggests that higher thresholds offered limited added value, as individuals taking nine or more medications likely share similarly elevated levels of risk. In contrast, lower thresholds (2+ to 8+) are more informative in identifying individuals with progressively increasing health needs. As a simple and data-accessible metric, a polypharmacy threshold of eight medications provided the best discrimination for both outcomes and may support the monitoring of trends and the development of public health initiatives. This is particularly relevant given that nearly one in four community-dwelling older adults in Québec use at least eight medications, and over 85% of them are exposed to at least one inappropriateness criteria (Campeau Calfat et al., 2024).

### Interpretation within the Context of the Literature

Few studies have examined the impact of polypharmacy on predicting both mortality and emergency visits (Chae et al., 2024; Choi et al., 2023). Those studies showed a positive association between polypharmacy, defined at a threshold of five medications, and both outcomes, mostly from a clinical perspective (Chae et al., 2024; Choi et al., 2023). A population-based study in Korea including 2,955,755 older adults (2016–2018) found a stronger association between polypharmacy (5+ medications for at least 90 consecutive days) and mortality (OR = 1.52; 95% CI 1.48–1.56) than with emergency visits (OR = 1.29; 95% CI 1.28–1.30), after adjusting for comorbidities, sex, age, and insurance type (Chae et al., 2024). This contrasts with our findings, which suggest a stronger association between polypharmacy and frequent emergency visits than with mortality. This discrepancy may stem from our focus on frequent emergency visits rather than single visits, which are associated with different individual characteristics (LaCalle & Rabin, 2010; Slankamenac et al., 2019). Another Korean study, including 55,228 hospitalized colorectal cancer survivors aged 65 or older (2003–2012), found similar associations between polypharmacy and both emergency visits (hazard ratio = 1.13; 95% CI 1.10–1.17) and mortality (hazard ratio = 1.14; 95% CI 1.11–1.18) (Choi et al., 2023). These estimates are lower than ours, likely due to additional adjustments for factors such as frailty and specific diseases. Both studies reported a stronger association when using a higher threshold of 10 or more medications (hyperpolypharmacy) which aligns with our results (Chae et al., 2024; Choi et al., 2023). Gnjidic et al. (2012) observed the predictive value of the number of medication used on one-year mortality among community-dwelling men aged 70 and older (*n* = 1,750) using questionnaire data from 2005 to 2007. Using the Younden Index, they identified medication thresholds of 6.5, 5.5, and 4.5 for predicting frailty, disability, and mortality, respectively, in models without covariates. In models adjusting for medication count, age, and medical conditions, the AUC values for these outcomes were 0.70, 0.62, and 0.61. Each additional medication was associated with a 9% increase in the odds of mortality (OR = 1.09; 95% CI 1.04–1.15) (Gnjidic et al., 2012). In our study, the adjusted odd ratio was of 1.06 (99% CI 1.05–1.06) which is coherent with Gnjidic et al. results. However, our model predicting mortality from a clinical perspective yielded a higher AUC of 0.80, compared to their 0.61. When testing various polypharmacy thresholds, we found nine medications best predicted mortality. This discrepancy may be explained by differences in study populations, including our younger sample, the inclusion of women, a healthier cohort, and the inclusion of additional covariates in all our analysis. Thus, our higher threshold may reflect greater resilience to medication adverse effects in healthier and more diverse populations.

### Strengths and Limitations

Our study evaluated simultaneously the predictive capacity of two measures of polypharmacy (count and threshold) and inappropriateness criteria (anticholinergic burden, PIMs, drug–drug interactions) on frequent emergency visits and one-year mortality. Those outcomes are important both from clinical and public health perspectives, and they are often measured using administrative databases. Measuring medication use through a variety of definitions enabled a broader and more comprehensive understanding of its potential impact on predicting frequent emergency visits and one-year mortality. Our population-based study reduces selection bias as we included almost all community-dwelling adults aged over 65 years in the province of Québec, Canada.

Our study shares the limitations of administrative databases. While we measured the dispensing of medications, we cannot guarantee their actual use on April 1, 2022, particularly if medications are taken only as needed. Therefore, the number of medications used may have been overestimated in some cases. On the other hand, medication use can be underestimated by the exclusion of over-the-counter medications and medications excluded from the public insurance plan. Although the number of diseases is easy to interpret, disease count does not fully capture the complexity of each condition. Furthermore, the number of diseases may be underestimated in administrative data (Yurkovich et al., 2015). In addition, our models did not include variables such as frailty that were not available in our database but which could have helped better understand the impact of polypharmacy on the prediction of outcomes. Although our results may be generalizable to other populations that share similar characteristics, caution is warranted, as the association between medication use and health outcomes is closely linked to health policy within each jurisdiction.

## Conclusion

Our findings suggest that a polypharmacy threshold of eight medications could guide public health surveillance. From a clinical perspective, while each additional medication increases the risk of adverse outcomes, the use of inappropriateness criteria may be a stronger measure of individual risk for frequent emergency visits and mortality. Although these results may not immediately warrant changes from a clinical perspective, they underscore the importance of considering polypharmacy as measure for public health interventions.

## Supplemental Material

Supplemental Material - Polypharmacy Thresholds that Best Predict Emergency Room Visits and Mortality in Older Adults: A Population-Based Study in Québec, Canada Supplemental Material for Polypharmacy Thresholds that Best Predict Emergency Room Visits and Mortality in Older Adults: A Population-Based Study in Québec, Canada by Alexandre Campeau Calfat, Justin P. Turner, Marc Simard, Marjolaine Dubé, Caroline Sirois in Journal of Applied Gerontology.

## Data Availability

The datasets generated and analyzed during the current study are not publicly available due to individual privacy stakes.*
