# Supplementary material for: Polypharmacy Thresholds that Best Predict Emergency Room Visits and Mortality in Older Adults: A Population-Based Study in Québec, Canada
Source: J Appl Gerontol. 2025 Oct 28;45(9):1809–21. doi: 10.1177/07334648251391524 (PMC13438002; doi:10.1177/07334648251391524)
Supplement: Supplementary file 1 [file sj-pdf-1-jag-10.1177_07334648251391524.pdf]

Polypharmacy thresholds that best predict emergency room visits and mortality in older adults: a population-based study in Québec, Canada

Supplementary File

Tables S1, S2 and S3 present the performance measures and association measures of models that use polypharmacy thresholds to predict both outcomes. Those models are from a clinical perspective and include disease count.

Tables S4 & S5 present the performance measures of models that use polypharmacy thresholds to predict both outcomes. Those models are from a public health perspective and exclude disease count.

Table S1: Performance measures of each model using polypharmacy thresholds to predict one-year frequent emergency visits (≥3) from a clinical perspective

			Reclassification Improvement		
**Model**	**Discrimination AUC (99% CI)**	**Overall Performance Scaled Brier Score (99% CI)**	**NRI (99% CI)**	**Frequent emergency visits percent improvement (%)**	**Nonfrequent emergency visits percent improvement (%)**
Baseline1	0.729 (0.726 - 0.731)	0.035 (0.033 - 0.036)	REF	REF	REF
Threshold2+	0.730 (0.727 - 0.732)	0.035 (0.033 - 0.036)	0.241 (0.234 - 0.249)	87.8	24.2
Threshold3+	0.731 (0.728 - 0.733)	0.035 (0.034 - 0.036)	0.342 (0.334 - 0.351)	83.3	33.8
Threshold4+	0.732 (0.729 - 0.735)	0.036 (0.034 - 0.037)	0.436 (0.428 - 0.445)	77.8	44.0
Threshold5+	0.734 (0.731 - 0.736)	0.036 (0.035 - 0.037)	0.507 (0.497 - 0.516)	71.4	54.0
Threshold6+	0.735 (0.732 - 0.738)	0.037 (0.035 - 0.038)	0.550 (0.540 - 0.559)	64.2	63.3
Threshold7+	0.736 (0.733 - 0.739)	0.038 (0.036 - 0.039)	0.557 (0.545 - 0.568)	56.6	71.3
Threshold8+	0.737 (0.734 - 0.739)	0.038 (0.037 - 0.039)	0.516 (0.503 - 0.529)	49.0	76.8
Threshold9+	0.737 (0.733 - 0.739)	0.039 (0.037 - 0.040)	0.431 (0.420 - 0.446)	42.1	79.5
Threshold10+	0.736 (0.733 - 0.739)	0.039 (0.038 - 0.041)	0.335 (0.317 - 0.352)	35.7	81.0
Threshold11+	0.736 (0.733 - 0.738)	0.040 (0.038 - 0.041)	0.230 (0.214 - 0.245)	30.3	81.1
Threshold12+	0.735 (0.732 - 0.737)	0.040 (0.038 - 0.041)	0.132 (0.115 - 0.150)	25.9	80.7
Threshold13+	0.734 (0.731 - 0.737)	0.040 (0.038 - 0.041)	0.053 (0.035 - 0.071)	22.6	80.0

Threshold14+	0.733 (0.730 - 0.736)	0.039 (0.038 - 0.041)	-0.013 (-0.037 - 0.010)	20.7	78.6
Threshold15+	0.732 (0.729 - 0.735)	0.039 (0.038 - 0.040)	-0.059 (-0.080 - -0.034)	19.8	77.3
Threshold16+	0.732 (0.729 - 0.734)	0.039 (0.037 - 0.040)	-0.080 (-0.113 - -0.052)	19.1	76.9
Threshold17+	0.731 (0.728 - 0.734)	0.038 (0.037 - 0.039)	-0.103 (-0.137 - -0.078)	19.6	75.2
Threshold18+	0.731 (0.727 - 0.733)	0.038 (0.036 - 0.039)	-0.107 (-0.141 - -0.076)	19.0	75.6
Threshold19+	0.730 (0.727 - 0.732)	0.037 (0.036 - 0.038)	-0.102 (-0.136 - -0.071)	19.6	75.3
Threshold20+	0.730 (0.727 - 0.732)	0.037 (0.035 - 0.038)	-0.105 (-0.142 - -0.069)	20.4	74.4

CI: confidence interval; AUC: Area Under Curve; NRI: Net Reclassification Improvement;

Predictors included in Baseline1 model: age, sex, social deprivation, material deprivation, zone, disease count

Table S2: Performance measures of each model using polypharmacy thresholds to predict one-year mortality from a clinical perspective					
			**Reclassification Improvement**		
**Model**	**Discrimination AUC (99% CI)**	**Overall Performance Scaled Brier Score (99% CI)**	**NRI (99% CI)**	**Mortality percent improvement (%)**	**Nonmortality percent improvement (%)**
Baseline2	0.800 (0.797 - 0.802)	0.052 (0.051 - 0.054)	REF	REF	REF
Threshold2+	0.800 (0.797 - 0.802)	0.052 (0.051 - 0.054)	0.251 (0.242 - 0.259)	89.0	23.6
Threshold3+	0.800 (0.797 - 0.803)	0.052 (0.051 - 0.054)	0.362 (0.352 - 0.371)	85.1	33.0
Threshold4+	0.801 (0.798 - 0.803)	0.052 (0.051 - 0.054)	0.463 (0.452 - 0.474)	80.1	43.0
Threshold5+	0.801 (0.799 - 0.804)	0.052 (0.051 - 0.054)	0.540 (0.529 - 0.551)	74.1	52.9
Threshold6+	0.802 (0.800 - 0.805)	0.053 (0.051 - 0.054)	0.587 (0.575 - 0.599)	67.3	62.1
Threshold7+	0.803 (0.800 - 0.805)	0.053 (0.051 - 0.054)	0.596 (0.583 - 0.609)	59.6	70.1
Threshold8+	0.803 (0.801 - 0.806)	0.053 (0.052 - 0.055)	0.551 (0.538 - 0.564)	51.8	75.8
Threshold9+	0.804 (0.801 - 0.806)	0.054 (0.052 - 0.055)	0.455 (0.441 - 0.472)	44.3	78.5
Threshold10+	0.803 (0.801 - 0.806)	0.054 (0.052 - 0.056)	0.345 (0.328 - 0.364)	37.1	80.2
Threshold11+	0.803 (0.801 - 0.806)	0.054 (0.053 - 0.056)	0.237 (0.217 - 0.257)	31.0	80.8
Threshold12+	0.803 (0.800 - 0.806)	0.055 (0.053 - 0.056)	0.146 (0.123 - 0.168)	26.3	81.0
Threshold13+	0.803 (0.800 - 0.806)	0.054 (0.053 - 0.056)	0.072 (0.050 - 0.096)	22.7	80.9
Threshold14+	0.802 (0.800 - 0.805)	0.054 (0.053 - 0.056)	0.026 (-0.007 - 0.052)	20.7	80.5
Threshold15+	0.802 (0.799 - 0.804)	0.054 (0.052 - 0.055)	-0.000 (-0.042 - 0.036)	19.5	80.5
Threshold16+	0.801 (0.799 - 0.804)	0.054 (0.052 - 0.055)	0.010 (-0.025 - 0.048)	19.4	81.1
Threshold17+	0.801 (0.799 - 0.804)	0.054 (0.052 - 0.055)	0.018 (-0.033 - 0.059)	20.1	80.8
Threshold18+	0.801 (0.798 - 0.803)	0.053 (0.052 - 0.055)	0.047 (-0.012 - 0.101)	19.7	82.8
Threshold19+	0.800 (0.798 - 0.803)	0.053 (0.052 - 0.055)	0.071 (0.021 - 0.116)	20.5	83.1
Threshold20+	0.800 (0.798 - 0.803)	0.053 (0.051 - 0.054)	0.093 (0.043 - 0.141)	20.9	83.8

CI: confidence interval; AUC: Area Under Curve; NRI: Net Reclassification Improvement;

Predictors included in Baseline2 model: age, sex, social deprivation, material deprivation, zone, disease count

Table S3: Odds ratio of frequent emergency visits and one-year mortality associated with medication use in each predictive model from a clinical perspective				
Model	Frequent emergency visit		Mortality	
	Odds ratio	99% CI	Odds ratio	99% CI
Threshold2+[⊥]	1.26	1.21 - 1.30	1.08	1.04 - 1.12
Threshold3+	1.34	1.30 - 1.38	1.17	1.13 - 1.22
Threshold4+	1.41	1.37 - 1.45	1.25	1.21 - 1.29
Threshold5+	1.49	1.45 - 1.53	1.31	1.27 - 1.35
Threshold6+	1.58	1.54 - 1.62	1.38	1.34 - 1.42
Threshold7+	1.64	1.60 - 1.68	1.42	1.38 - 1.46
Threshold8+	1.70	1.66 - 1.74	1.47	1.43 - 1.51
Threshold9+	1.75	1.71 - 1.80	1.51	1.47 - 1.56
Threshold10+	1.81	1.77 - 1.86	1.55	1.50 - 1.59
Threshold11+	1.88	1.83 - 1.93	1.61	1.56 - 1.66
Threshold12+	1.96	1.90 - 2.01	1.69	1.63 - 1.74
Threshold13+	2.05	1.98 - 2.11	1.74	1.68 - 1.80
Threshold14+	2.15	2.07 - 2.22	1.77	1.70 - 1.85
Threshold15+	2.30	2.21 - 2.39	1.83	1.75 - 1.91
Threshold16+	2.48	2.37 - 2.60	1.94	1.84 - 2.04
Threshold17+	2.62	2.48 - 2.77	2.04	1.92 - 2.17
Threshold18+	2.76	2.59 - 2.95	2.20	2.04 - 2.36
Threshold19+	2.97	2.75 - 3.21	2.30	2.10 - 2.51
Threshold20+	3.21	2.92 - 3.53	2.43	2.18 - 2.71
RxCount*	1.08	1.08 - 1.08	1.06	1.05 - 1.06
ACB*	1.16	1.15 - 1.16	1.17	1.16 - 1.18
PIM*	1.26	1.25 - 1.28	1.18	1.16 - 1.20
Intx*	1.52	1.49 - 1.55	1.51	1.48 - 1.55

*Estimated increase in risk for each number added.

[⊥]Estimated increase in risk when using an equal or higher number of medication than the threshold.

CI: confidence interval; ACB: anticholinergic cognitive burden; PIM: potentially inappropriate medication; Intx: drug-drug interaction

Predictors included in model: age, sex, social deprivation, material deprivation, zone, disease count

**Table S4: Performance measures of each model used to predict one-year frequent emergency visits (≥3) from a public health perspective**					
			**Reclassification Improvement**		
**Model**	**Discrimination AUC (99% CI)**	**Overall Performance Scaled Brier Score (99% CI)**	**NRI (99% CI)**	**Frequent emergency visits percent improvement (%)**	**Nonfrequent emergency visits percent improvement (%)**
Baseline3	0.633 (0.630 - 0.636)	0.010 (0.010 - 0.011)	REF	REF	REF
Threshold2+	0.649 (0.646 - 0.652)	0.012 (0.012 - 0.013)	0.247 (0.241 - 0.253)	88.2	24.1
Threshold3+	0.658 (0.655 - 0.661)	0.014 (0.013 - 0.014)	0.346 (0.338 - 0.354)	83.6	33.7
Threshold4+	0.668 (0.666 - 0.671)	0.015 (0.015 - 0.016)	0.437 (0.428 - 0.445)	77.9	44.0
Threshold5+	0.678 (0.675 - 0.680)	0.017 (0.017 - 0.018)	0.507 (0.497 - 0.516)	71.4	54.0
Threshold6+	0.685 (0.682 - 0.688)	0.019 (0.019 - 0.020)	0.550 (0.540 - 0.559)	64.2	63.3
Threshold7+	0.689 (0.686 - 0.692)	0.021 (0.020 - 0.022)	0.557 (0.548 - 0.568)	56.5	71.3
Threshold8+	0.690 (0.687 - 0.693)	0.022 (0.021 - 0.023)	0.536 (0.526 - 0.547)	48.7	78.1
Threshold9+	0.688 (0.685 - 0.691)	0.023 (0.022 - 0.024)	0.494 (0.484 - 0.505)	41.2	83.6
Threshold10+	0.684 (0.681 - 0.687)	0.023 (0.022 - 0.024)	0.438 (0.428 - 0.448)	34.1	87.9
Threshold11+	0.679 (0.676 - 0.682)	0.023 (0.022 - 0.024)	0.375 (0.366 - 0.385)	27.6	91.2
Threshold12+	0.673 (0.670 - 0.676)	0.022 (0.021 - 0.023)	0.311 (0.302 - 0.319)	21.8	93.8
Threshold13+	0.667 (0.664 - 0.670)	0.021 (0.020 - 0.022)	0.251 (0.243 - 0.258)	16.9	95.6
Threshold14+	0.661 (0.658 - 0.664)	0.020 (0.019 - 0.021)	0.197 (0.190 - 0.204)	12.9	97.0
Threshold15+	0.656 (0.653 - 0.659)	0.019 (0.018 - 0.020)	0.154 (0.148 - 0.160)	9.7	98.0
Threshold16+	0.651 (0.648 - 0.654)	0.018 (0.017 - 0.019)	0.117 (0.111 - 0.122)	7.2	98.7
Threshold17+	0.647 (0.643 - 0.650)	0.016 (0.015 - 0.017)	0.085 (0.080 - 0.089)	5.1	99.1
Threshold18+	0.644 (0.640 - 0.646)	0.015 (0.014 - 0.016)	0.060 (0.056 - 0.063)	3.6	99.4
Threshold19+	0.641 (0.637 - 0.644)	0.014 (0.013 - 0.015)	0.043 (0.039 - 0.050)	2.6	99.6
Threshold20+	0.639 (0.635 - 0.642)	0.013 (0.012 - 0.014)	0.036 (0.028 - 0.045)	2.4	99.4

CI: confidence interval

Predictors included in Baseline3 model: age, sex, social deprivation, material deprivation, zone

Table S5: Performance measures of each model used to predict one-year mortality from a public health perspective					
			Reclassification Improvement		
**Model**	**Discrimination AUC (99% CI)**	**Overall Performance Scaled Brier Score (99% CI)**	**NRI (99% CI)**	**Mortality percent improvement (%)**	**Nonmortality percent improvement (%)**
Baseline4	0.731 (0.728 - 0.735)	0.031 (0.030 - 0.032)	REF	REF	REF
Threshold2+	0.738 (0.735 - 0.741)	0.031 (0.030 - 0.033)	0.251 (0.242 - 0.259)	89.0	23.6
Threshold3+	0.743 (0.740 - 0.746)	0.032 (0.031 - 0.033)	0.362 (0.352 - 0.371)	85.1	33.0
Threshold4+	0.749 (0.746 - 0.752)	0.033 (0.032 - 0.035)	0.463 (0.452 - 0.474)	80.1	43.0
Threshold5+	0.755 (0.752 - 0.758)	0.034 (0.033 - 0.036)	0.540 (0.529 - 0.551)	74.1	52.9
Threshold6+	0.760 (0.757 - 0.763)	0.036 (0.035 - 0.037)	0.587 (0.575 - 0.599)	67.3	62.1
Threshold7+	0.763 (0.760 - 0.766)	0.037 (0.036 - 0.038)	0.596 (0.585 - 0.609)	59.6	70.1
Threshold8+	0.764 (0.761 - 0.767)	0.038 (0.037 - 0.039)	0.572 (0.560 - 0.585)	51.7	76.9
Threshold9+	0.764 (0.761 - 0.767)	0.038 (0.037 - 0.040)	0.526 (0.513 - 0.538)	43.8	82.5
Threshold10+	0.762 (0.759 - 0.765)	0.038 (0.037 - 0.040)	0.461 (0.448 - 0.472)	36.2	86.9
Threshold11+	0.759 (0.756 - 0.762)	0.038 (0.037 - 0.040)	0.394 (0.382 - 0.404)	29.3	90.4
Threshold12+	0.756 (0.753 - 0.759)	0.038 (0.036 - 0.039)	0.325 (0.315 - 0.334)	23.2	93.1
Threshold13+	0.753 (0.750 - 0.756)	0.037 (0.035 - 0.038)	0.257 (0.246 - 0.266)	17.8	95.1
Threshold14+	0.749 (0.746 - 0.752)	0.035 (0.034 - 0.037)	0.195 (0.185 - 0.202)	13.2	96.6
Threshold15+	0.745 (0.742 - 0.749)	0.035 (0.033 - 0.036)	0.146 (0.138 - 0.152)	9.7	97.6
Threshold16+	0.742 (0.739 - 0.746)	0.034 (0.033 - 0.036)	0.108 (0.101 - 0.114)	7.0	98.4
Threshold17+	0.740 (0.737 - 0.743)	0.033 (0.032 - 0.035)	0.077 (0.071 - 0.082)	4.9	98.9
Threshold18+	0.738 (0.735 - 0.741)	0.033 (0.032 - 0.034)	0.055 (0.051 - 0.059)	3.5	99.3
Threshold19+	0.736 (0.733 - 0.739)	0.032 (0.031 - 0.034)	0.038 (0.034 - 0.048)	2.3	99.5
Threshold20+	0.735 (0.732 - 0.738)	0.032 (0.031 - 0.033)	0.032 (0.022 - 0.055)	2.1	99.6

CI: confidence interval

Predictors included in Baseline4 model: age, sex, social deprivation, material deprivation, zone
